# The Impact of Surgical Correction of Neuromuscular Scoliosis on Respiratory Muscle Function in Individuals with Spinal Muscular Atrophy—Preliminary Report

**DOI:** 10.3390/jcm15041615

**Published:** 2026-02-19

**Authors:** Edyta Daszkiewicz, Barbara Jasiewicz, Karina Rożek, Piotr Kurzeja, Michał Zarzycki, Zbigniew Figura, Aleksandra Adamik, Katarzyna Ogrodzka-Ciechanowicz

**Affiliations:** 1 Department of Orthopaedics and Rehabilitation, University Hospital of Orthopaedics Rehabilitation in Zakopane, Jagiellonian University Collegium Medicum, 31-008 Krakow, Poland; edyta.daszkiewicz@uj.edu.pl (E.D.); barbara.jasiewicz@uj.edu.pl (B.J.); michal.zarzycki@uj.edu.pl (M.Z.); zbigniew.figura@uj.edu.pl (Z.F.); 2 Institute of Social Sciences and Public Health, Pedagogical University of Krakow, 30-084 Krakow, Poland; karina.rozek@uken.krakow.pl; 3 Institute of Health Sciences, University of Applied Sciences in Nowy Targ, 34-400 Nowy Targ, Poland; piotrkurzeja@op.pl; 4 Institute of Applied Sciences, Faculty of Motor Rehabilitation, University of Physical Culture, 31-571 Krakow, Poland; aleksandra.adamik@awf.krakow.pl; 5 Institute of Clinical Rehabilitation, Faculty of Motor Rehabilitation, University of Physical Culture, 31-571 Krakow, Poland

**Keywords:** spinal muscular atrophy, idiopathic scoliosis, maximum inspiratory pressure, maximum expiratory pressure

## Abstract

**Background:** The aim of this prospective longitudinal observational study was to assess respiratory muscle function after surgical correction of scoliosis in individuals with spinal muscular atrophy (SMA). **Material**: The study included 20 patients (aged 7–19) with scoliosis in the course of neuromuscular disease, eligible for surgical treatment with diagnosed SMA Type 2 or 3. **Methods**: Measurements were taken of the maximal inspiratory pressure (MIP) [cmH_2_O] and the maximal expiratory pressure (MEP) [cmH_2_O] in all patients immediately before surgical correction of scoliosis (measurement I), 7 days after surgery (measurement II) and 3 months after surgery (measurement III). **Results**: The mean Cobb angle of spinal curvature measured before surgery in the entire group was 102.57 ± 17.96. The mean MEP values in the entire group of patients were 40.48 ± 11.76 cmH_2_O before surgery, 36.74 ± 17.17 cmH_2_O after 7 days, and 39.17 ± 16.18 cmH_2_O 3 months after surgery. The MIP values for the entire group were 64.35 ± 28.40 cmH_2_O before surgery, 53.96 ± 28.66 cmH_2_O after 7 days, and 67.00 ± 31.27 cmH_2_O after 3 months. **Conclusions**: Surgical correction of spinal curvature creates conditions for maintaining respiratory muscle strength in patients with SMA over a period of several months of observation. As a result of the surgical intervention, respiratory muscle strength did not deteriorate, but even slightly increased.

## 1. Introduction

Spinal muscular atrophy (SMA) is a genetic disease that causes muscle weakness and atrophy in infants and children, and less commonly in adults [1]. The incidence of SMA is approximately 1 in 10,000 to 20,000 live births, and the carrier frequency is 1/40 to 1/70 in the general population [2]. SMA is considered to be the leading genetic cause of infant mortality [3].

SMA is currently divided into five subtypes: Type 0 (the most severe form, which begins in the prenatal period and develops into severe breathing problems after birth), Type 1 (Werdnig–Hoffmann disease—a severe form that begins before the age of 6 months and prevents independent sitting), Type 2 (Dubowitz disease—an intermediate form that begins before the age of 18 months and prevents independent sitting, standing and walking), Type 3 (Kugelberg–Welanders disease—a mild form that begins after the age of 18 months and prevents independent standing and walking) and Type 4 (the mildest form, which begins after the age of 30 years) [4].

SMA Types 1–3 frequently feature complications, including scoliosis and joint contractures. The onset of scoliosis exacerbates difficulties in daily functioning, particularly in patients who are unable to sit or walk independently [5]. The frequency of contractures increases with the progression of SMA and occurs in joints that are important for mobility, such as knees, hips, and elbows [5]. Currently, there is a growing consensus that it would be appropriate to reclassify patients with SMA, taking into account the spectrum of the disease and the current clinical condition, including mobility, especially with regard to the emergence of disease-modifying treatments [5].

The development of spinal deformities in SMA and other neuromuscular disorders results from weakness of axial muscles and insufficient support for the growing spine. Spinal deformities are considered a significant complication of these disorders [6]. Patients with SMA typically have hypotonic spinal curvature, which can result in progressive scoliosis at an early age, particularly in SMA Types 1 and 2 [6,7]. Scoliosis can significantly affect motor function and negatively impact respiratory function. A large percentage of patients with SMA develop scoliosis, the onset and severity of which are usually related to the severity of the SMA subtype [7,8]. In SMA, scoliosis most commonly occurs in the thoracolumbar spine in a C-shape or, less frequently, in an S-shape, often accompanied by pelvic obliquity and increased thoracic kyphosis [7,8,9]. Given the altered shape of the torso and reduced rib mobility, these changes can seriously affect sitting ability and respiratory function [9].

The natural course of scoliosis in SMA initially shows a gradual increase in spinal curvature, progressing at 7.2 degrees (Cobb angle) per year. This is followed by a period of rapid progression of 10.2 degrees per year during the 18 months prior to surgery [7]. The progression of curvature is slower in milder subtypes of SMA [7,8]. The likelihood of requiring scoliosis surgery is approximately 80% in individuals with SMA Types 1c and 2 [7,8].

Scoliosis, as a three-dimensional deformity of the spine, but also of the trunk, can have negative consequences in terms of self-perception and can cause pain, as well as possible negative effects requiring surgical or therapeutic treatment [10]. In paediatric patients with SMA, deterioration of axial muscle strength during growth spurts can lead to spinal collapse and, consequently, significant scoliosis at a young age. Consequently, severe scoliosis in childhood affects the development and functioning of the cardiopulmonary system of patients, as well as trunk balance [9,11]. It is well known that bone deformities of the spine and chest accompanying scoliosis may be associated with restrictive pulmonary ventilation impairment. The natural history of SMA scoliosis shows that during adolescence, the process of disease development intensifies and becomes not only an orthopaedic but also a pulmonary problem. Impaired lung function can manifest itself even with little physical exertion. A progressive stiffening of deformed tissues further restricts mobility of the chest. All of this leads to a reduction in the total lung capacity and its component parts—including vital capacity and residual volume. Reduction in lung size leads to exclusion of some alveoli from breathing and, consequently, to reduced lung compliance. These changes disrupt gas exchange by increasing the ratio of ventilation to blood flow in the lungs. The energetic cost of breathing is also increased, caused directly by deformation of the chest [12]. Patients with scoliosis require special care and regular orthopaedic checkups throughout their growth period, as well as systematic assessment of respiratory function [13]. Indirect assessment of respiratory muscle strength appears particularly important, especially in the context of planned surgical treatment for scoliosis. Efficient function of these muscles is essential for maintaining proper alveolar ventilation both at rest and under changing conditions of health and disease.

Because the forces generated by the respiratory muscles cannot be directly measured within the respiratory system, their function is assessed using indirect methods [14]. The simplest test is the measurement of vital capacity (VC), which to some extent reflects the mobility of the thorax and abdomen. However, this test is not fully specific, as VC is influenced by many factors not directly related to respiratory muscle strength. However, normal VC values generally exclude significant weakness of these muscles [14,15].

A more precise assessment of respiratory muscle strength is possible by measuring the maximum static pressures generated in the respiratory system, namely the maximum inspiratory pressure (MIP) and the maximum expiratory pressure (MEP) [14,16]. In children with scoliosis, a factor contributing to respiratory muscle dysfunction is chest deformation, which further limits mobility.

In clinical practice, it is important to incorporate breathing exercises into the comprehensive rehabilitation of patients with spinal muscular atrophy (SMA) after surgical treatment of scoliosis. Surgical correction remains the most effective method for preventing hypoxia in scoliosis. However, no studies have been published to date assessing respiratory muscle strength in patients with SMA undergoing surgical treatment of scoliosis.

In light of the above, the purpose of the study was to assess respiratory muscle function after surgical correction of scoliosis in patients with SMA.

## 2. Materials and Methods

### 2.1. Study Design

This is a prospective longitudinal observational study. It was conducted in accordance with the principles of the Declaration of Helsinki and the STROBE Statement (Strengthening the Reporting of Observational Studies in Epidemiology) guidelines for reporting observational studies [17]. The project received approval from the Bioethics Committee of the Jagiellonian University (no. 1072.6120.137.2022) on 31 August 2022. Before enrolment in the study, all participants provided written, informed consent.

### 2.2. Setting

The study included 25 patients from the University Orthopaedic and Rehabilitation Hospital of the Jagiellonian University Medical College in Zakopane, who were subjected to surgical treatment for idiopathic scoliosis between 2022 and 2025.

### 2.3. Participants

The first author enrolled 25 patients, who were patients of the University Orthopaedic and Rehabilitation Hospital. Each patient received information about the purpose of the project, the measurement equipment, and the principles of data confidentiality and presentation, and written consent for the children to participate in the study was collected.

Inclusion criteria:

1. Scoliosis in the course of neuromuscular disease (spinal muscular atrophy); idiopathic juvenile scoliosis diagnosed by an orthopaedist (based on medical history and X-ray examination with Cobb angle measurement) eligible for surgical treatment in children with SMA Type 2 or 3; SMA diagnosis made by paediatric neurologists based on genetic testing.
2. Age ≥ 7 years;
3. No other concomitant diseases that could affect the outcome of the study;
4. Written consent of a parent (guardian) to participate in the study.

**Figure 1:**
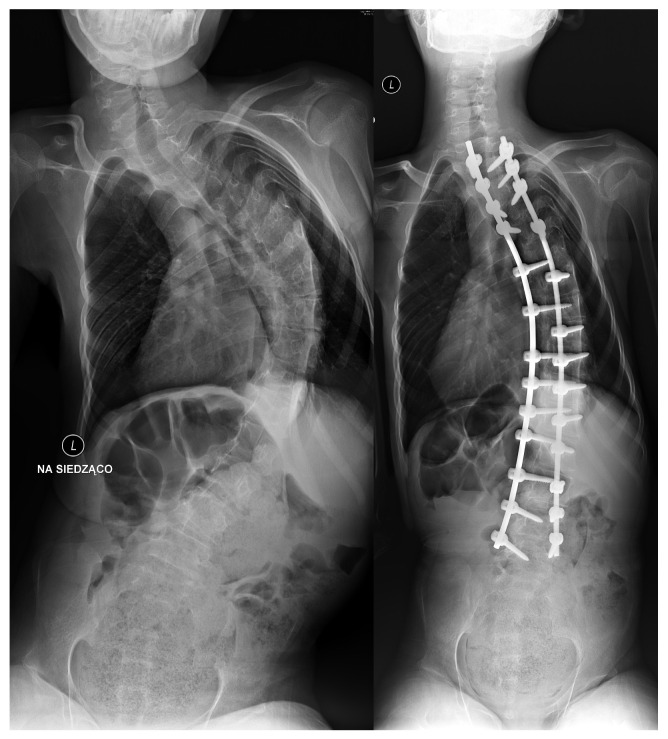
X-ray of the spine before and after surgical correction of scoliosis. A 13-year-old boy with thoracolumbar scoliosis. AP X-ray before and after surgery [own source].

The group consisted of 20 individuals, of whom 8 had SMA Type 3 and 12 had SMA Type 2. All children had scoliosis, and the difference between the types was related to the age at diagnosis. Patients underwent scoliosis correction surgery with a posterior approach: deformity correction with long spinal fusion. The average blood loss during the procedure was 302.60 mL (±148.08 mL). The ICU stay averaged 3 days (min–max: 1–4). Patients were extubated after the surgery. In the ICU, they were on spontaneous breathing with oxygen therapy or on assisted ventilation (including their own equipment used the night before surgery). Medications included: fluids, electrolytes, blood (if needed), pain medications (Figure 1).

### 2.4. Outcome Measures

The maximal inspiratory pressure (MIP) and maximal expiratory pressure were measured using a MicroRPM digital respiratory muscle strength meter manufactured by Micro Medical (MICRO-MEDICAL Instrumente GmbH, Senftenberg, Germany).

MicroRPM is a portable device designed to assess respiratory muscle strength. The meter measures maximal inspiratory and expiratory pressures through the mouth (MIP/MEP) and sniff nasal inspiratory pressure (SNIP). The MicroRPM meter uses a piezoresistive sensor to measure pressure during inhalation and exhalation, ensuring high accuracy and long-term stability of measurements. Specialised electronic circuits calculate the pressure and display the result in cmH_2_O. The measuring range of the device is between −25 kPa and +25 kPa. The measurement error does not exceed 0.5% of the upper limit of the measurement range. The measurement outcome is displayed on a digital readout panel, including the sign of the measured pressure (relative to atmospheric pressure), with a resolution of 0.1 kPa. Both pressures are measured within the same measuring system upon prior deletion of the measurement result obtained in the previous experiment. In the clinical context, the evaluation of the strength of respiratory muscles is performed with measurements of maximum inspiratory pressure (MIP) and maximum expiratory pressure (MEP) exerted through the mouth [18,19]. It has recently been shown that -MEP/-MIP ratio could be a suitable and straightforward parameter for establishing with certainty strength loss in respiratory muscles [20,21].

### 2.5. Intervention

Measurements were taken of the maximal inspiratory pressure (MIP) [cmH_2_O] and the maximal expiratory pressure (MEP) [cmH_2_O] in all patients immediately before surgical correction of scoliosis (measurement I), 7 days after surgery (measurement II) and 3 months after surgery (measurement III). All measurements were made by the same person. Pulmonary function measurements were performed 7 days after surgery to assess the early impact of the surgical intervention on the respiratory system and to identify any ventilation disturbances related to postoperative pain, limited chest mobility, and convalescence. Measurements performed 3 months after surgery were aimed at assessing mid-term respiratory adaptation, stabilisation of results, and the degree of improvement or recovery of pulmonary function after the early phase of postoperative rehabilitation.

MIP was measured at residual volume, and the MIP value was taken as the pressure that the subject was able to maintain for 1 s.

MEP was measured at the level of total lung capacity. The subjects performed an inhalation or exhalation exercise with their airways blocked, and the MIP/MEP values were recorded after 1 s.

Both pressures were measured using the same measuring system after deleting the measurement result obtained in the previous experiment. The measurement error did not exceed 0.2% in this range for a measurement range of 50 kPa. Tests were performed in a sitting position and consisted of 3 to 5 trials. The highest values obtained were treated as maximum values if at least one other result differed from the maximum by no more than 10%.

### 2.6. Statistical Analysis

Statistical analyses were performed using Statistica 13.3 (StatSoft, Tulsa, OK, USA). Descriptive statistics were calculated for all variables and presented as means, standard deviations, medians, and ranges. The normality of distribution was assessed using the Shapiro–Wilk test. Because the study design involved repeated measurements in the same participants (before surgery, 7 days after surgery, and 3 months after surgery), changes over time were primarily analysed using repeated-measures analysis of variance (ANOVA) for normally distributed variables. When the assumptions of normality were not met, appropriate nonparametric tests were applied. Between-group comparisons (girls vs. boys) were performed using independent-samples *t*-tests or the Mann–Whitney U test, depending on data distribution. Post hoc tests (Tukey) were used when significant overall effects were detected. In addition to *p*-values, effect sizes were calculated to improve the interpretability of the results. For overall time effects, Kendall’s coefficient of concordance (W) was reported. For pairwise comparisons between time points, Cohen’s d was calculated based on group means and standard deviations. Effect sizes were interpreted as small (0.2), moderate (0.5), and large (0.8). The level of statistical significance was set at *p* < 0.05.

## 3. Results

**Figure 2:**
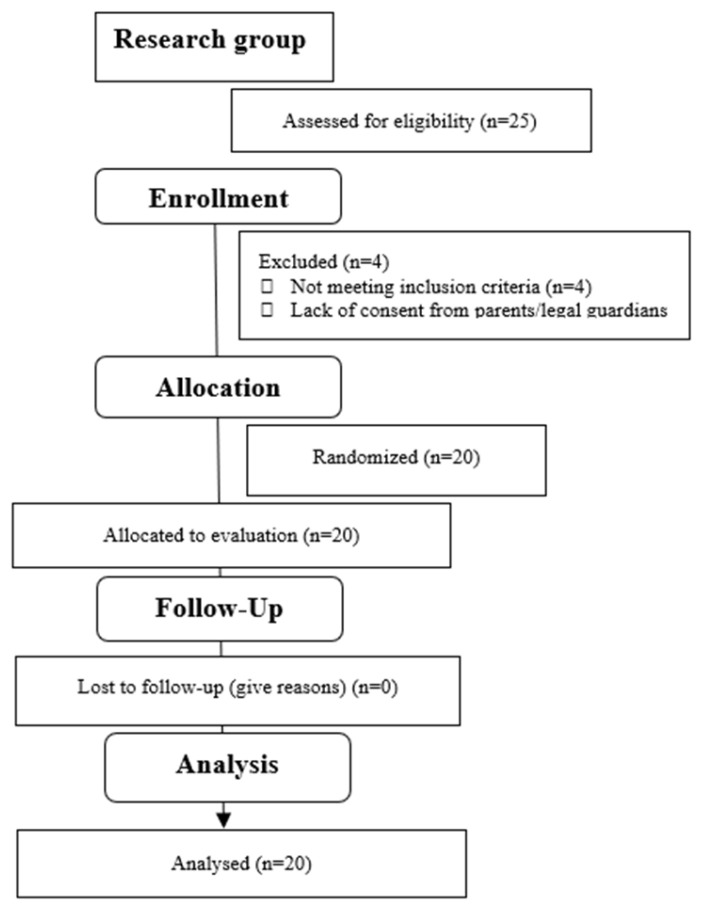
Flow diagram.

Twenty-five patients aged 7–19 years were enrolled in the study. Due to written refusals of some parents/guardians to allow their children to participate in the study, and failure to meet the inclusion criteria, the final stage of the study included 20 individuals (8 girls, 12 boys). The mean age of the subjects was 13.74 ± 5.05 years (range 7–19 years). The girls were slightly younger (12.09 ± 2.21 years) than the boys (15.25 ± 6.43 years), but this difference was not statistically significant (*p* = 0.14). The mean body weight in the entire group was 37.83 ± 12.59 kg and did not differ significantly between the sexes (girls—37.36 ± 12.04 kg; boys—38.25 ± 13.60 kg; *p* = 0.87). The duration of surgery was 166.30 ± 35.84 min on average, with boys tending to have longer procedures (179.58 ± 27.51 min) than girls (151.82 ± 39.89 min); this difference was borderline statistically significant (*p* = 0.06). A comparable group structure was obtained in terms of age and body weight, which allowed for further functional analyses without the need for demographic adjustment. Detailed data on age, body weight and duration of surgery in the study groups are presented in Table 1. The enrolment stage is presented in Figure 2.

The mean Cobb angle before surgery in the entire group was 102.57 ± 17.96. The values were similar in girls (102.55 ± 19.98°) and boys (102.58 ± 16.80°), and the differences between the sexes were not statistically significant (*p* = 0.99). After surgery, a significant improvement was achieved: the mean Cobb angle decreased to 50.74 ± 21.86°, corresponding to a reduction in curvature of approximately 52° (*p* < 0.001 within groups). In both the girls’ group (*p* < 0.001) and the boys’ group (*p* < 0.001), the differences between preoperative and postoperative measurements were statistically significant, while no significant differences between the sexes were observed in terms of the correction achieved (*p* = 0.79). This indicates comparable effectiveness of the procedure in both groups (Table 2).

The mean MEP values in the entire group of patients were 40.48 ± 11.76 cmH_2_O before surgery, 36.74 ± 17.17 cmH_2_O after 7 days, and 39.17 ± 16.18 cmH_2_O 3 months after surgery. The analysis of variance (ANOVA) showed statistical significance for the entire group (*p* = 0.04), but pairwise comparisons (d2-1, d3-1, d3-2) revealed no significant differences (*p* > 0.05). This indicates a slight, temporary postoperative decrease in expiratory muscle strength, which stabilised at baseline levels within 3 months. No significant differences between sexes were found at any stage of measurement (*p* = 0.14; 0.83; 0.69).

The MIP values for the entire group were 64.35 ± 28.40 cmH_2_O before surgery, 53.96 ± 28.66 cmH_2_O after 7 days, and 67.00 ± 31.27 cmH_2_O after 3 months. The ANOVA showed significant changes over time (*p* < 0.001). A significant decrease in MIP was observed immediately after the procedure (*p* = 0.04 in girls; *p* = 0.08 in boys), while after 3 months, a renewed increase in values was observed, exceeding the baseline level (*p* = 0.03 in girls; *p* = 0.01 in boys). No significant differences were found between sexes (*p* = 0.32; 0.88; 0.38).

For expiratory muscle strength (MEP), only slight fluctuations were observed, with no significant long-term differences.

Detailed values are presented in Table 3.

The analysis of changes in maximal inspiratory pressure (MIP) between successive measurements showed that on the 7th day after the surgery, MIP values were lower compared to the preoperative measurement. The mean difference for the entire group was −10.39 ± 30.25 cmH_2_O (range from −76 to 82 cmH_2_O; Me = −14.00). In the girls’ group, this decrease averaged −14.18 ± 19.97 cmH_2_O, while in boys it was −6.92 ± 37.95 cmH_2_O. The differences between sexes did not reach statistical significance (*p* = 0.41).

Three months after the procedure, the mean change in MIP from baseline was positive at +2.65 ± 29.18 cmH_2_O (range from −48 to 92 cmH_2_O; Me = 0.00). In the girls’ group, the mean increase was +1.55 ± 22.02 cmH_2_O, and in the boys it was +3.67 ± 35.50 cmH_2_O; yet again, no significant differences between the sexes were found (*p* = 0.78).

The results obtained indicate that after surgical correction of scoliosis, there is a short-term decrease in inspiratory muscle strength in the first week after the procedure, while after three months, MIP values return to baseline or slightly exceed it.

Detailed values are presented in Table 4.

Analysis of changes in maximal expiratory pressure (MEP) in subsequent stages of the study showed that 7 days after the scoliosis correction surgery, MEP values were slightly lower compared to preoperative measurements.

The mean difference for the entire group was −3.74 ± 11.05 cmH_2_O (range from −27 to 19 cmH_2_O; Me = −2.00). A very slight decrease (−0.82 ± 10.16 cmH_2_O) was observed in the group of girls, while the decrease was slightly greater in boys (−6.42 ± 11.57 cmH_2_O). The differences between the sexes were not statistically significant (*p* = 0.31).

Three months after surgery, the mean change in MEP values relative to baseline was not statistically significant at −1.30 ± 11.57 cmH_2_O (range from −30 to 24 cmH_2_O; Me = 0.00). A slight increase (+2.73 ± 10.69 cmH_2_O) was observed in the group of girls, while a slight decrease (−5.00 ± 11.53 cmH_2_O) was observed in the group of boys. The differences between the sexes also did not reach statistical significance (*p* = 0.14). (Table 5.)

### Effect Size Analysis

Effect size analysis demonstrated a moderate overall time effect for MIP (Kendall’s W = 0.32) and a small effect for MEP (W = 0.12). Pairwise comparisons showed a small-to-moderate short-term decrease in MIP 7 days after surgery (Cohen’s d = 0.36), followed by a moderate increase between 7 days and 3 months (d = 0.43). Long-term changes between the preoperative and 3-month measurements were negligible for both MIP (d = 0.09) and MEP (d = 0.09). Changes in MEP across time were small (d = 0.15–0.26). (Table 6.)

## 4. Discussion

In their pilot study, the authors indirectly assessed respiratory muscle strength in 20 individuals undergoing surgical treatment for idiopathic scoliosis with SMA Type 2 or 3. The mean Cobb angle of spinal curvature measured before surgery in the entire group was 102.57 ± 17.96. All patients were subject to measurement of maximal inspiratory pressure (MIP) [cmH_2_O] and maximal expiratory pressure (MEP) [cmH_2_O] immediately before surgical correction of scoliosis (measurement 1), 7 days after surgery (measurement 2) and 3 months after surgery (measurement 3). The results indicate that after surgical correction of scoliosis, there is a short-term decrease in inspiratory muscle strength in the first week after surgery, while after three months, MIP values return to baseline or slightly exceed it. Similar trends were observed for maximal expiratory pressure (MEP).

Scoliosis remains very common in children with SMA Type 1 and 2, with a prevalence of 60–90% in early childhood [3,22]. The size of hypotonic spinal curvatures steadily increases during childhood, and most patients also develop excessive thoracic kyphosis of varying severity. As a consequence of weak trunk and chest muscle support, children with SMA are more likely to develop chest bone and joint disorders as a result of developing scoliosis and progressive chest deformity [23,24].

Maintaining proper respiratory function requires adequate chest volume and proper functioning of the diaphragm and internal and external intercostal muscles. Scoliosis often causes deformities of the spine and chest, resulting in compression of the lung tissue, imbalance of the intercostal muscles on both sides, decreased respiratory muscle strength, and reduced compliance of the chest wall and lungs, which affects lung ventilation function [25].

As a result of the progression of the disease, surgical treatment of the spine is often indicated in order to maintain the balance of the torso in a sitting position, realign the deformed chest to improve respiratory function, and improve overall quality of life [26,27]. The decision to perform spinal surgery is based primarily on the size of the curvature (i.e., Cobb angle of the main curvature ≥50°) and the rate of progression (≥10° per year). Account should also be taken of other factors such as decreased respiratory function, rib deformity, hyperkyphosis, and adverse effects on functional mobility, pelvic obliquity and trunk imbalance. Pulmonary function tests should be considered as part of the preoperative assessment to determine the surgical and postoperative risk to respiratory function [23,26,27,28].

The available literature refers to studies that assessed the natural course of lung dysfunction in patients with SMA; however, FVC was most commonly used to assess respiratory function in patients with SMA [29,30,31,32,33,34,35]. The studies observed a progressive decline in FVC caused by increasing respiratory muscle failure, limited lung and chest wall growth, and progression of scoliosis [36,37].

Respiratory muscle strength may decline earlier than lung volume in neuromuscular diseases, and for this reason, assessment of respiratory muscle strength is sometimes used more frequently than measurements of vital capacity to detect weakness in these muscles [38,39,40,41,42]. MIP and MEP are commonly used to assess lung function in patients with AIS [43]. MIP and MEP can detect respiratory failure earlier than FVC because they are likely to be related to muscle denervation.

Spirometry is the most widely used test for assessing lung volume and capacity over time. Due to its importance in the diagnosis and treatment of respiratory disorders, spirometry is included in the recommendations for the care of numerous neuromuscular diseases [44,45,46]. Ginanneschi et al. [47] demonstrated a progressive decline in MIP, MEP and FVC values over 11 years of follow-up in patients with SMA. The authors demonstrated a correlation between the three spirometric indices and disease progression, suggesting that clinical decline is closely related to worsening spirometric data. The decline in MEP and MIP values was severe, while that in FVC was mild.

Fabian et al. found that MIP, MEP and FVC values in patients with scoliosis are lower compared to healthy adolescents, and FVC values correlated significantly with respiratory muscle strength, suggesting that these patients had previously experienced mild pulmonary dysfunction and that early intervention was necessary to prevent further deterioration and reduce its impact on adolescent health [48].

Lin et al. [49] confirmed that in the idiopathic scoliosis group, lung function correlates mainly with the angle of scoliosis, the location of the curvature and the age of patients. However, these authors concluded that there is no single factor (test) that reflects lung function in scoliosis.

Jasiewicz et al. assessed respiratory muscle strength before and after surgery for idiopathic scoliosis [50]. The studies examined respiratory muscle strength, taking into account the type of surgery (anterior and posterior access). In both groups of patients, the differences between measurements before surgery and 7 days after surgery were statistically significant (*p* = 0.011). However, a higher maximal inspiratory pressure was recorded in the group after posterior access surgery. According to the authors, this indicates that anterior access surgery may be associated with a higher risk of negative effects on respiratory muscle function, especially in the short term after surgery.

Lung function in idiopathic scoliosis before and after surgery has been studied by multiple authors [50,51,52,53,54,55,56]. The vast majority of the authors cited pointed to a deterioration in spirometric lung function in children after surgical correction of idiopathic scoliosis; however, the authors did not analyse maximal inspiratory and expiratory pressures. Some believed that postoperative deterioration in pulmonary function test results should be taken into account in the preoperative assessment of postoperative risk [57].

Taking the above into account, the authors of this study decided to focus on the assessment of indirect respiratory muscle strength in patients with SMA who were subjected to surgery for scoliosis. It is also important to note that no such studies were found in the available literature.

Saito et al. [58] analysed the effect of surgical correction of spinal curvature on respiratory muscle strength in 16 patients with Duchenne muscular dystrophy (DMD). The results indicated that scoliosis correction in patients with DMD may have a beneficial effect on respiratory muscle function.

Flores et al. [59] assessed respiratory muscle strength in 12 girls qualified for surgery due to juvenile idiopathic scoliosis, in whom the Cobb angle of spinal curvature ranged from 42° to 62°. Significantly lower MIP and MEP values were observed compared to controls without scoliosis. The authors suggested that MIP and MEP values below 30 cmH_2_O increase the risk of postoperative respiratory failure; therefore, an appropriate respiratory muscle strength training programme was recommended for these patients in order to potentially reduce the risk of postoperative complications.

Pulmonary function test results provide an important basis for planning and modifying the postoperative rehabilitation process. Measurements taken early after surgery enable the assessment of the degree of lung ventilation limitation, which allows for the adjustment of exercise intensity, the introduction of appropriate breathing techniques, and the prevention of respiratory complications. Results obtained three months after surgery allow for the assessment of the effectiveness of the rehabilitation programme, the degree of respiratory adaptation, and the planning of further rehabilitation steps, including increasing workload and functional activity in a safe and individually tailored manner.

Optimal respiratory care for patients with SMA requires multidisciplinary paediatric care, including neurologists, pulmonologists and physiotherapists. Systematic and regular assessment of respiratory muscle function is crucial for the prevention and effective treatment of respiratory complications in these children.

### Study Limitations

As the study concerned a rare disease treated surgically for scoliosis, the main limitation is the small sample size compared to other publications describing lung function disorders and respiratory muscle strength assessment in other clinical cases. The authors are aware that they did not fully assess lung function in their patients. In the next stage of the study, they intend to analyse the respiratory system more extensively, extending the diagnosis to include detailed spirometry and body plethysmography. Further limitations are: absence of a non-surgical control group, potential learning effects in repeated MIP/MEP testing, and the relatively short follow-up period. Analysis of the results of the study showed that immediately after surgery, scoliosis correction is a factor that reduces respiratory muscle strength in patients with SMA. However, the benefits of improved spinal biomechanics after correction consist in maintaining the initial muscle strength, with the prospect of strengthening within a few months after the procedure.

## 5. Conclusions

Surgical correction of spinal curvature created conditions for maintaining respiratory muscle strength in patients with SMA over a period of several months of observation. As a result of the surgical intervention, respiratory muscle strength did not deteriorate, but even slightly increased. It seems that properly planned preoperative and postoperative rehabilitation, taking into account pulmonary disorders, is an important element of rehabilitation for these patients, having a significant impact on quality of life in the early postoperative period. The results are preliminary and generate hypotheses. The authors are continuing similar studies in which the assessment of respiratory muscle function will also be conducted one year after surgery and will be extended to include spirometry tests.

## Figures and Tables

**Table 1:** Characteristics of the patients who took part in the study.

	Girls			Boys			All			
	$\bar{X}$ ± SD	Min–Max	Me	$\bar{X}$ ± SD	Min–Max	Me	$\bar{X}$ ± SD	Min–Max	Me	*p*-Value mg
Age	12.09 ± 2.21	9.00–16.00	12.00	15.25 ± 6.43	7.00–19.00	13.00	13.74 ± 5.05	7.00–19.00	12.00	0.14
Body weight	37.36 ± 12.04	19.00–5.00	35.00	38.25 ± 13.60	20.00–75.00	36.00	37.83 ± 12.59	19.00–75.00	35.00	0.87
Duration of surgery	151.82 ± 39.89	100.00–55.00	145.00	179.58 ± 27.51	130.00–225.00	187.50	166.30 ± 35.84	100.00–55.00	165.00	0.06

*p* mg—statistical significance between groups; $\bar{X}$ —arithmetic mean; SD—standard deviation; Me—median; Min–Max—minimum and maximum values; *p*-value—significance of differences between groups.

**Table 2:** Cobb angle before and after scoliosis correction surgery in the study group of patients.

Indicators	Girls			Boys			All			*p* mg
	$\bar{X}$ ± SD	Min–Max	Me	$\bar{X}$ ± SD	Min–Max	Me	$\bar{X}$ ± SD	Min–Max	Me	
Cobb before	102.55 ± 19.98	67.00–124.00	110.00	102.58 ± 16.80	76.00–140.00	101.00	102.57 ± 17.96	67.00–140.00	102.00	0.99
Cobb after	49.82 ± 25.29	22.00–113.00	40.00	51.58 ± 19.31	23.00–95.00	52.50	50.74 ± 21.86	22.00–113.00	50.00	0.85
*p* wg	0.00 *			0.00 *			0.00 *			
Diff Cobb	52.73 ± 21.59	9.00- 76.00	57.00	51.00 ± 7.57	35.00–3.00	53.00	51.83 ± 15.53	9.00–76.00	53.00	0.79

Cobb before—Cobb angle value before surgery; Cobb after—Cobb angle value after surgery; Diff Cobb—difference in Cobb angle value before and after surgery; *p* wg—intragroup statistical significance; *p* mg—intergroup statistical significance (<0.05); *—statistically significant.

**Table 3:** Changes in maximal inspiratory pressure (MIP) and expiratory pressure (MEP) at three time points.

	Group	Before (1)			After (2)			3 mth After (3)				*p*-Value		
		$\bar{X}$ ± SD	SE	Min–Max	$\bar{X}$ ± SD	SE	Min– Max	$\bar{X}$ ± SD	SE	Min– Max	ANOVA	d 2-1	d 3-1	d 3-2
MEP	Girls	36.36 ± 7.57	2.28	25.00– 49.00	35.55 ± 13.57	4.09	17.00–63.00	39.09 ± 13.69	4.12	13.00–64.00	0.36	0.79	0.42	0.24
	Boys	44.25 ± 13.86	4.00	22.00– 66.00	37.83 ± 20.47	5.91	14.00–68.00	39.25 ± 18.79	5.42	18.00–71.00	0.11	0.16	0.16	0.76
	All	40.48 ± 11.76	2.45	22.00– 66.00	36.74 ± 17.17	3.58	14.00–68.00	39.17 ± 16.18	3.37	13.00–71.00	0.04 *	0.12	0.59	0.37
	*p*-Value	0.14			0.83			0.69						
MIP	Girls	66.91 ± 21.07	6.35	44.00–103.00	52.73 ± 24.07	7.26	21.00–90.00	68.45 ± 22.67	6.84	21.00–113.00	0.03 *	0.04 *	0.82	0.03 *
	Boys	62.00 ± 34.60	9.98	30.00–160.00	42.00 ± 33.36	9.63	16.00–130.00	65.67 ± 38.53	11.12	30.00–140.00	0.08	0.73	0.73	0.01 *
	All	64.35 ± 28.40	5.92	30.00–160.00	53.96 ± 28.66	5.98	16.00–130.00	67.00 ± 31.27	6.52	21.00–140.00	0.00 *	0.67	0.67	0.00 *
	*p*-Value	0.32			0.88			0.38						

*—statistically significant.

**Table 4:** Differences in maximal inspiratory pressure (Diff MIP) between preoperative measurements, measurements 7 days after surgery, and measurements 3 months after scoliosis correction surgery.

		$\bar{X}$ ± SD	Me	Min–Max
Diff MIP after–before	Girls	−14.18 ± 19.97	−16.00	−36.00–31.00
	Boys	−6.92 ± 37.95	−13.00	−76.00–82.00
	All	−10.39 ± 30.25	−14.00	−76.00–82.00
	*p*-Value ANOVA	0.41		
Diff MIP 3 mth after–before	Girls	1.55 ± 22.02	1.00	−33.00–36.00
	Boys	3.67 ± 35.50	0.00	−48.00–92.00
	All	2.65 ± 29.18	0.00	−48.00–29.19
	*p*-Value ANOVA	0.78		

Diff MIP after–before—MIP difference before and after surgery; Diff MIP 3 mth after–before—MIP difference before and 3 months after surgery; $\bar{X}$—arithmetic mean; SD—standard deviation; Me—median; Min–Max—minimum and maximum values; *p*-value—statistical significance (<0.05).

**Table 5:** Differences in maximal expiratory pressure (Diff MEP) between preoperative measurements, measurements 7 days after surgery, and measurements 3 months after scoliosis correction surgery.

		$\bar{X}$ ± SD	Me	Min–Max
Diff MEP after–before	Girls	−0.82 ± 10.16	−2.00	−19.00–19.00
	Boys	−6.42 ± 11.57	−4.50	−27.00–8.00
	All	−3.74 ± 11.05	−2.00	−27.00–19.00
	*p*-Value ANOVA	0.31		
Diff MEP 3 mth after–before	Girls	2.73 ± 10.69	1.00	−14.00–24.00
	Boys	−5.00 ± 11.53	−3.50	−30.00–9.00
	All	−1.30 ± 11.57	0.00	−30.00–24.00
	*p*-Value ANOVA	0.14		

Diff MEP after–before—MEP difference before and after surgery; Diff MEP 3 mth after–before—MEP difference before and 3 months after surgery; $\bar{X}$—arithmetic mean; SD—standard deviation; Me—median; Min–Max—minimum and maximum values; *p*-value—statistical significance (<0.05).

**Table 6:** Effect sizes for changes in maximal inspiratory pressure (MIP) and maximal expiratory pressure (MEP) over time in the entire study group.

Variable	Comparison	Effect Size	Interpretation
Global effect			
MIP	Time (1-2-3)	W = 0.32	Moderate
MEP	Time (1-2-3)	W = 0.12	Small
Pairwise comparisons (Cohen’s d)			
MIP	1–2 (before vs. 7 days)	0.36	Small–moderate
MIP	1–3 (before vs. 3 months)	0.09	Negligible
MIP	2–3 (7 days vs. 3 months)	0.43	Moderate
MEP	1–2 (before vs. 7 days)	0.26	Small
MEP	1–3 (before vs. 3 months)	0.09	Negligible
MEP	2–3 (7 days vs. 3 months)	0.15	Very small

Effect sizes were calculated based on group means and standard deviations.

## Data Availability

The minimal data set is contained within our paper.
